# *Plasmodium vivax* Cysteine-Rich Protective Antigen Polymorphism at Exon-1 Shows Recombination and Signatures of Balancing Selection

**DOI:** 10.3390/genes12010029

**Published:** 2020-12-28

**Authors:** Lilia González-Cerón, José Cebrián-Carmona, Concepción M. Mesa-Valle, Federico García-Maroto, Frida Santillán-Valenzuela, Jose Antonio Garrido-Cardenas

**Affiliations:** 1 Centro Regional de Investigación en Salud Pública, Instituto Nacional de Salud Pública, Tapachula 30700, Chiapas, Mexico; fsantill@insp.mx; 2 Departamento de Biología y Geología, Universidad de Almería, 04120 Almería, Spain; jcc851@inlumine.ual.es (J.C.-C.); cmesa@ual.es (C.M.M.-V.); 3 Departamento de Química y Física, Universidad de Almería, 04120 Almería, Spain; fgmaroto@ual.es

**Keywords:** *Plasmodium vivax*, *pvcyrpa*, Southern Mexico, Cysteine-Rich Protective Antigen (CyRPA), merozoite protein, genetic diversity, Tajima’s D, MK test, phylogenetic tree, balancing selection

## Abstract

*Plasmodium vivax* Cysteine-Rich Protective Antigen (CyRPA) is a merozoite protein participating in the parasite invasion of human reticulocytes. During natural *P. vivax* infection, antibody responses against PvCyRPA have been detected. In children, low anti-CyRPA antibody titers correlated with clinical protection, which suggests this protein as a potential vaccine candidate. This work analyzed the genetic and amino acid diversity of *pvcyrpa* in Mexican and global parasites. Consensus coding sequences of *pvcyrpa* were obtained from seven isolates. Other sequences were extracted from a repository. Maximum likelihood phylogenetic trees, genetic diversity parameters, linkage disequilibrium (LD), and neutrality tests were analyzed, and the potential amino acid polymorphism participation in B-cell epitopes was investigated. In 22 sequences from Southern Mexico, two synonymous and 21 nonsynonymous mutations defined nine private haplotypes. These parasites had the highest LD-R^2^ index and the lowest nucleotide diversity compared to isolates from South America or Asia. The nucleotide diversity and Tajima’s D values varied across the coding gene. The exon-1 sequence had greater diversity and Rm values than those of exon-2. Exon-1 had significant positive values for Tajima’s D, β-α values, and for the Z (HA: dN > dS) and MK tests. These patterns were similar for parasites of different origin. The polymorphic amino acid residues at PvCyRPA resembled the conformational B-cell peptides reported in PfCyRPA. Diversity at *pvcyrpa* exon-1 is caused by mutation and recombination. This seems to be maintained by balancing selection, likely due to selective immune pressure, all of which merit further study.

## 1. Introduction

Malaria is a disease that affects millions of people worldwide. In the Americas, *P. vivax* is widely distributed and the number of cases and severity of the disease has increased in recent years [1]. Malaria vaccines using merozoite proteins aim to reduce parasitemia and, consequently, the disease’s severity [2]. This approach eventually reduces transmission at the regional level, which is necessary to achieve malaria elimination [3]. Furthermore, merozoite proteins that induce antibody responses are useful markers for serological surveillance [4,5].

For blood infection to occur, *Plasmodium* merozoite invasion requires multiple and sequential interactions between the protein ligands and the erythrocyte surface receptors. In *Plasmodium falciparum*, the cysteine-rich protective antigen (CyRPA) has been studied more than in other *Plasmodium* species. PfCyRPA forms a complex with other proteins and is essential in the sequential molecular events leading to the merozoite invasion of human erythrocytes [6]. PfCyRPA is capable of binding to the reticulocyte-binding homolog 5 protein (PfRH5) and the RH5-interacting protein (PfRipr). The PfRH5/PfRIPR/CyRPA complex is highly immunogenic, making these proteins promising vaccine components against malarial blood stages [7]. The PfCyRPA protein sequence has been highly conserved among global parasite strains; in exposed individuals from Ghana, anti-PfCyRPA antibodies were not detected [7]. In *P. falciparum*, anti-CyRPA-specific antibodies substantially inhibited erythrocyte invasion by merozoites in in vitro culture and in an in vivo NOD-scid IL2Rγ (null) mouse model grafted with human erythrocytes [8]. Recent studies have shown the high immunogenicity of PfCyRPA delivered in virosomes to immunized mice and rabbits. In addition, the IgG from immunized rabbits reduced parasite development to 77% in a *P. falciparum* infection mouse model [9]. 

In the *P. knowlesi* and *P. vivax* genomes, the RH5 coding gene is not present. In *P. knowlesi*, the RIPR and CyRPA proteins do not seem to form a complex with each other; rather RIPR forms a novel trimeric protein complex with other molecules, whereas CyRPA, as a single protein, is essential for merozoite invasion [10]. Knuepfer et al. [10] showed that erythrocyte receptor basigin (BSG) might not be essential for erythrocyte invasion in *P. knowlesi*, as it is in *P. falciparum* [7]. 

In *P. vivax*, the *cyrpa* gene consists of two exons and one intron located on chromosome five, encoding for a microneme protein. Anti-BSG receptor antibodies caused different growth inhibition rates in field isolates and suggested that BSG might be an alternative receptor on the reticulocyte for merozoite invasion [10]. Unlike in *P. falciparum*, *P. vivax* CyRPA seems to be exposed to the immune system. In young children, CyRPA induced high IgG antibody levels against *P. vivax* during the infection, and low antibody titers were strongly associated with protection from the clinical disease [11]. 

To understand the potential of PvCyRPA in vaccine development and/or as a serological marker, the nucleotide and amino acid diversity, and the natural selection acting on this molecule were studied in symptomatic patients in Southern Mexico and compared to parasites from other geographical origins. The polymorphism of the peptide regions that potentially participate as B-cell epitopes was explored. Homologous sequences of different origins were retrieved from a repository and included in the analysis. Our results show that *pvcyrpa* had lower nucleotide diversity in parasites from Southern Mexico than in parasites from South America or Asia. This diversity has been shaped by mutation and recombination, and it has probably been maintained by balancing selection. The pattern of nucleotide diversity across the coding gene was consistent in parasites of different origin, with exon-1 showing a greater number of nonsynonymous mutations than exon-2. The neutrality and selection tests suggest that *pvcyrpa* exon-1 is probably under positive balancing selection pressure; accordingly, the amino acid variation was detected in peptide regions predicted as being B-cell epitopes. These findings suggest that recombination and immune selective pressure played a role in shaping PvCyRPA diversity. 

## 2. Materials and Methods

The Ethics Committee of the Mexican National Institute of Public Health project (CI1042) approved the study, and informed consent was obtained from the patients. All the samples analyzed were obtained from adults (over 18 years of age).

### 2.1. Samples and Origin

Ten blood samples infected with *P. vivax* were selected for this study, obtained between 2000 and 2015 from the Pacific side of Southern Chiapas, Mexico (Southern Mexico). In this region, after implementing intensive and simultaneous measures based on vector control and patient management, *P. falciparum* was eliminated late in the 1990s while *P. vivax* transmission remained. From 2000 onwards, the number of malaria cases decreased gradually over time. Mexico entered the pre-elimination phase in 2007 [12]. Most patients from the region experienced an uncomplicated disease [13]. In symptomatic patients showing no severe symptoms and seeking a malaria diagnosis, *P. vivax* was diagnosed via microscopy using a thick Giemsa-stained smear. Capillary blood samples from finger pricks were used to impregnate Whatman #2 filter paper. Afterwards, the patients were treated in accordance with the Mexican guidelines for malaria treatment, and the administration of treatments based on chloroquine and primaquine was supervised by the malaria control program officials [14]. Dried *P. vivax* blood samples were kept at 4 °C in the dark prior to use.

### 2.2. DNA Extraction, PCR Amplification and Sequencing

Genomic DNA from whole infected blood samples was extracted using the GeneJET Whole Blood Genomic DNA Purification Mini Kit (Thermo Fisher Scientific, Asheville, NC 28803, USA), as indicated by the manufacturer. *P. vivax* single infection was verified using species-specific oligonucleotides (Table 1), and the conditions for the nested PCR using the *UNR*, *PLF* and *VIR* primers were as described by Rubio et al. [15]. To obtain and sequence the *pvcyrpa* coding gene, nine primers were designed (Table 1). The IDT Company (Integrated DNA Technologies, Inc., Coralville, IA 52241, USA) synthesized the oligonucleotides.

CYRPA R1, F1, R2, and F2 primers were designed from the sequencing of several *P. vivax* strains retrieved from available databases, while ClustalW in Bioedit v7.0.5.3 [16,17] was used for DNA alignment. The internal primers CYRPA_R3, F3, R4, F4, and R5 were designed to complete the DNA coding sequence. The first amplification was performed with the SensiFAST™ PCR Kit (Bioline, Cincinnati, OH 45244, USA) in a final volume of 20 µL, using 200 ng of the whole genomic in a reaction containing the buffer (supplied by the manufacturer) and 5 ng of each of the primers. The PCR conditions consisted of a denaturation step in a MyGo Pro thermal cycler (Biocompare, Inc., Kansas City, MO 66103, USA) at 94 °C for 5 min, followed by 40 cycles, each at 94 °C for 45 s, 61 °C for 45 s, and 72 °C for 60 s, with a final extension at 72 °C for 10 min. Then, a nested PCR was performed using 0.1 µL of the previous PCR product with a denaturation step of 94 °C for 5 min, followed by 40 cycles at 94 °C for 30 s, 60 °C for 30 s, and 72 °C for 30 s, and the final extension at 72 °C for 10 min. The expected molecular size of the amplified products was verified using 1% agarose gels and visualized in a transilluminator.

The PCR products were purified using the mi-PCR Purification Kit (Metabion, 82152 Planegg, Germany) according to the manufacturer’s instructions. Afterwards, 5–50 ng of DNA were used per sequencing reaction employing the Sanger method, using forward and reverse primers in the ABI PRISM^®^ 3500 Genetic Analyzer System (Applied Biosystems, Carlsbad, CA 92008, USA); this was carried out at the nucleic acid analysis service of the Central Research Services at the University of Almeria, Spain.

The quality of the pherograms was revised manually, and using ClustalW in BioEdit, including Sal I sequence XM_001615090.1 [18] as the reference. No pherograms with double peaks were detected, which suggests that there was more than one genotype in the amplified DNA fragments. This coincided with previous work using *Pvama1_I-II_*, which showed a very low proportion of mixed genotype infections from 2000 to 2011 in Southern Mexico [19]. Numerous alignments were run using forward and reverse sequences to obtain the consensus sequences of the complete *pvcyrpa* coding genes. The sequences were deposited in the GenBank database, identified with the accession numbers MW010262–MW010268.

### 2.3. Gene Sequences

Seven sequences of the *pvcyrpa* coding gene were obtained in this study. Other homologous sequences from the same region (Southern Mexico) and from other geographic sites, were retrieved from a repository (https://plasmodb.org/plasmo/; *n* = 82) and included in the analysis (Table S1). For Southern Mexico, there were 22 coding sequences in total, comprising seven sequences from this study and 15 from the repository [20]. All the sequences were from parasites obtained in Southern Mexico (Figure S1). Two other sequence groups were prepared. The South American group consisted of 45 sequences (21 and 24 sequences of isolates from Colombia and Peru, respectively), while the Asian group comprised 19 sequences (9, 4, and 6 sequences of isolates from Thailand, Papua New Guinea and China, respectively). Three reference sequences were included. Since *pvcyrpa* has an intron, only complete sequences (Table S1) were used to estimate the LD indexes (Table S1). 

### 2.4. Genetic Analysis of the Coding Gene

To search for genetic similarities among haplotypes from Southern Mexico, and from around the world, maximal likelihood (ML) phylogenetic trees were constructed using the bootstrap method and 1000 replications in MEGA v6 [21]. Reference sequences from strains Sal I (XM_001615090.1) and from Belem and P01 (PvP01_0532400) were included. 

The genetic diversity parameters and the neutrality tests were estimated using sequences from Southern Mexico and from other geographic sites with higher transmission intensities, namely South America and Asia. The nucleotide diversity distribution across the *pvcyrpa* sequence was analyzed using slide window analysis of 100 bp fragments with 25 overlapping nucleotide units in DnaSP [22]. The number of haplotypes (H), the nucleotide (π) and genetic diversity (θ), the haplotype diversity, and the minimal number of recombination events (Rm) were calculated in DnaSP. 

To determine the departure from neutrality of the *cyrpa* coding gene, different tests were evaluated. The allele-frequency-based Tajima’s D test was estimated using slide window analysis per 100 bp and 25 overlapping nucleotides, and for the gene segments in DnaSP. Fu and Li’s D* and F* tests were estimated—the D* test analyzes differences between the number of singletons and the total number of mutations while the F* test computes the differences between the number of singletons and the average number of nucleotide differences [23]. These tests inform about the selection and demographic forces acting on a population. Positive values might be suggestive of positive or balancing selection (if other selection tests are significantly positive). This force maintains alleles at balanced frequencies. Conversely, negative values suggest purifying selection or recent population expansion [24]. 

To detect codons under positive selection across *pvcyrpa* coding gene, synonymous and nonsynonymous rates (α, β) were calculated using Fast Unconstrained Bayesian AppRoximation (FUBAR) in a website software (https://www.datamonkey.org/). This model uses a probabilistic approach to infer whether the nonsynonymous substitution rate (β) in a site is faster or slower than the neutral rate, which is set to the synonymous rate (α) at the same site. Probability, *p*, <0.05 detected very low false-positive rates (0.5%) on the neutral and purifying sites [25]. The codon-based Z test can detect if positive selection is operating on a gene fragment by comparing the relative numbers of non-synonymous versus synonymous substitutions and their variances [26]. The alternate hypothesis (HA: dN > dS) was tested using the Modified Nei-Gojobori method (Jukes-Cantor) with 1000 bootstrap replications in MEGA v6.; this was done because some genes that code for antigenic proteins, and that are under strong balancing selection, might produce positive skews in the McDonald and Kreitman (MK) divergence test, as shown for malarial blood-stage antigens [27]. To corroborate that recurrent natural selection is acting on the *cyrpa* coding sequence, the MK neutrality index [28] was calculated. The MK test is based on a comparison of synonymous and nonsynonymous variations within and between species. For this, we used the orthologous sequence of *Plasmodium cynomolgi* strain B (XM_004221254) [29], a species closely related to *P. vivax* [30], and the analysis was computed in DnaSP. This sequence shows higher (76.8%) protein identity with PvP01 (BlastP, NBCI). 

### 2.5. Linkage Disequilibrium (LD) Analysis and Mutations in the Intron

Sequences containing the complete gene were used to estimate the degree of non-random association between the haplotypes and at all the informative sites. The D’ and R^2^ indices of LD were calculated in DnaSP. 

### 2.6. Exploration of Potential Residues Participating in B-cell Epitopes in CYRPA Amino Acid Sequences of P. vivax From Southern Mexico

Peptides likely to contain B-cell epitopes were sought using the BepiPred method, which combines the hidden Markov model with one of the best propensity scale methods for predicting linear B-cell epitopes (https://www.iedb.org/) [31,32]. A threshold of 0.35, which comprises 75% specificity and 49% sensitivity, was used. The residues above the threshold are predicted to be part of an epitope. In addition, the accessibility scale was estimated for hexapeptides—if the Sn were above 1.0, this increased the probability that they were on the protein surface [33]. The *P. falciparum cyrpa* sequence (http://plasmodb.org; PF3D7_0423800) was used to make comparisons.

## 3. Results

### 3.1. Pvcyrpa Polymorphism at the Coding Gene of Parasites from Southern Mexico

The Sal I sequence and the seven sequences from this study had codons 14-17 (CTC TTC TCC TTC) at the 5’end. These codons seem to correspond to the signal peptide (4). There was no information on this segment in the sequences obtained from PlasmoDB; therefore, it could not be included in the genetic analysis. Twenty-two nucleotide coding sequences from Southern Mexico (each sequence was obtained from different *P. vivax* isolate), had two synonymous and 21 nonsynonymous mutations, and resolved nine haplotypes. Codon 271, present in the Sal I sequence, was only present in two divergent haplotypes (h2 and h9) in Southern Mexico (Table 2). 

Most mutations present in the *pvcyrpa* coding gene of the Southern Mexico parasites were also common in the parasites from South America and Asia (Table S2). Few mutations were less distributed or private; for example, mutations at codons 139 and 326 were shared between Southern Mexico and Colombia, present in three and two isolates, respectively. The mutation at codon 363 was present in one isolate from Southern Mexico, and present in a single isolate from Thailand. The mutation at codon 82 was exclusive to one isolate (h7) from Southern Mexico. Of the 57 haplotypes detected globally (n = 89), only two haplotypes were shared between Peru and Colombia, all the other haplotypes were private including those resolved for Southern Mexico. 

**Figure 1:**
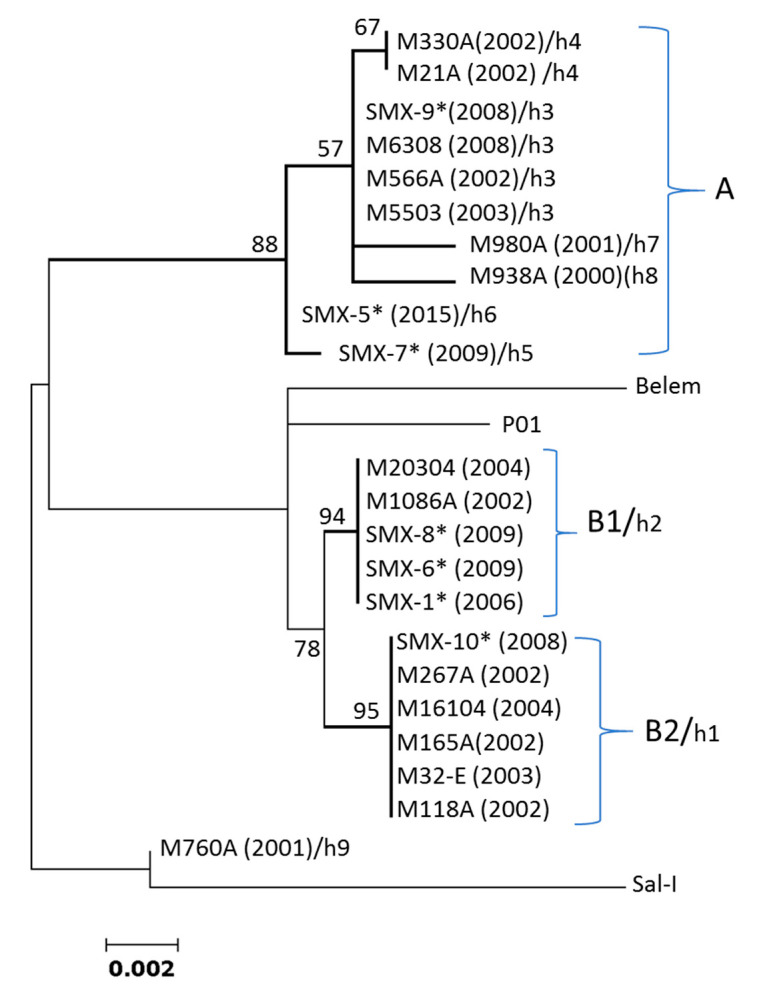
ML phylogenetic tree of the *P. vivax cyrpa* coding gene for 22 Southern Mexican parasites. Bootstraps above 50% are indicated. Sal I (XM_001615090.1), Belem, and PvP01 (PlasmoDB) were included as reference strains. These M760A isolate sequences did not cluster with other Southern Mexican parasites. The haplotype numbers h1–h9 assigned to each parasite isolate (as in Table 1) are indicated. * indicate sequences obtained in this study. (Year of sample collection).

The ML tree using the *pvcyrpa* coding gene demarcated two main clusters (A and B) in parasites from Southern Mexico, separated from each other by 88% and 78% bootstrap values, respectively; both were separated from isolate M760A and the reference sequences of the Sal I, Belem and P01 strains. Furthermore, haplotypes h1 and h2 from Southern Mexico in cluster B were separated by bootstrap value >90% (Figure 1). The global ML tree showed a lack of clustering by region or country. Haplotypes from the same or different regions were randomly scattered across the tree, and bootstrapping values above 50% were not observed in the primary or secondary external branches (Figure S2).

### 3.2. Sequence Diversity of the Pvcyrpa Coding Gene 

**Figure 2:**
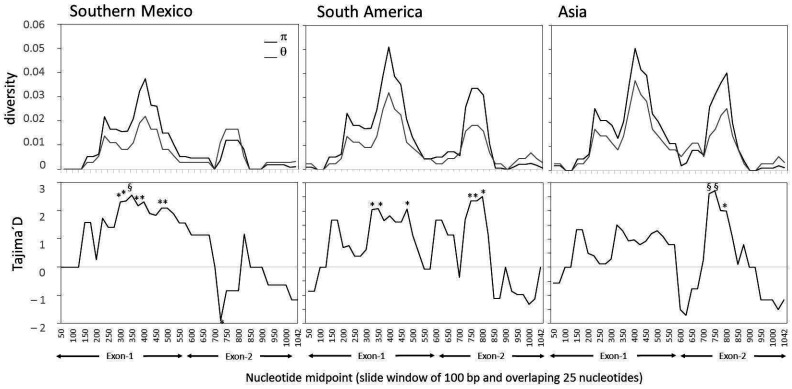
Slide window analysis of the diversity and Tajima’s D for the *P. vivax cyrpa* coding gene. Indexes of nucleotide (π) and genetic diversity (θ); Tajima’s D values varied across the sequence. Graphs show isolates from different geographical origin. Window length of 100 bp and step size of 25 nucleotides. Significance * *P <* 0.05; § *P <* 0.01.

In *P. vivax* from Southern Mexico, the slide window analysis showed that nucleotide (π) and genetic (θ) diversity were heterogeneously distributed across the coding region, and values were higher in exon-1 than in exon-2 (Figure 2). The pattern of diversity detected in Southern Mexico was similar to that of parasites from South America and Asia (Figure 2). 

Using the whole coding gene confirmed that Southern Mexico parasites were less diverse than parasites from other geographic origins (Table 3). For southern Mexican parasites, the nucleotide (t = 29.619, df = 65, *P* < 0.0001 unpaired t test) and haplotype (t = 16.80, df = 65, *P <* 0.0001) diversity were lower than those detected in South America, and, in turn, those in South America had lower nucleotide (t = 10.863, df = 62, *P* < 0.0001) and haplotype (t = 0 5.451, df = 62, *P* < 0.0001) diversity than parasites from Asia (Table 3). Similar differences were detected when analyzing each exon separately. The exon-1 sequence showed higher nucleotide diversity than that of exon-2, evidenced by the π and θ values, which were similar for all the parasite groups (Table 3). The nucleotide diversity of South America and Asian parasites was higher in exon-1 than exon-2 (t = 52.07, df = 88 and t = 19.443, df = 36 respectively; *P <* 0.0001). 

In contrast, for Southern Mexican parasites the haplotype diversity was higher in the exon-2 sequence than that of exon-1 (t = 10.08, df = 42, *P <* 0.0001; 95% CI). For South American parasites, the Hd for the exon-1 sequence was slightly higher than that in exon-2 (t = 2.067, df = 88, *P =* 0.0416), while the Asia group showed no difference in the Hd values between exons (t = 1.521, df = 36, *P =* 0.1370).

### 3.3. Neutrality Tests

The sliding window analysis of Tajima’s D (TjD) showed significant positive values in fragments of 100 bp positioned between the 200–500 nucleotides of exon-1 and a negative value at the 700 nucleotide midpoints (exon-2) in parasites from Southern Mexico (Figure 2). Parasite isolates from SA and Asia produced a different TjD-value pattern. Parasites from South America had significant positive values at exon-1, similar to the Southern Mexico parasites, and additional significant values between fragments comprising the 700–850 nucleotides of exon-2 (Figure 2). Conversely, in Asian parasites, the TjD values were positive along exon-1 but none were significant. At exon-2, positive significant values were obtained, similar to those in South America parasites (Figure 2).

The exon-1 analysis showed consistently positive TjD or Fu and Li’D* and/or Fu*and Li’F* values (Table 4). For isolates from Southern Mexico, the TjD and TjD (nonsynonymous) were significantly positive; for South American parasites, both the Fu and Li’D* and the Fu and Li’F* tests were significantly positive; and for the Asian parasites, only the Fu and Li’D* test was significant (Table 4). In Southern Mexico, the TjD, Fu and Li’D* and Fu and Li’F* tests showed negative values for exon-2 but were not significant, while for South America and Asia, the values were around 1.0 and near zero, respectively.

**Figure 3:**
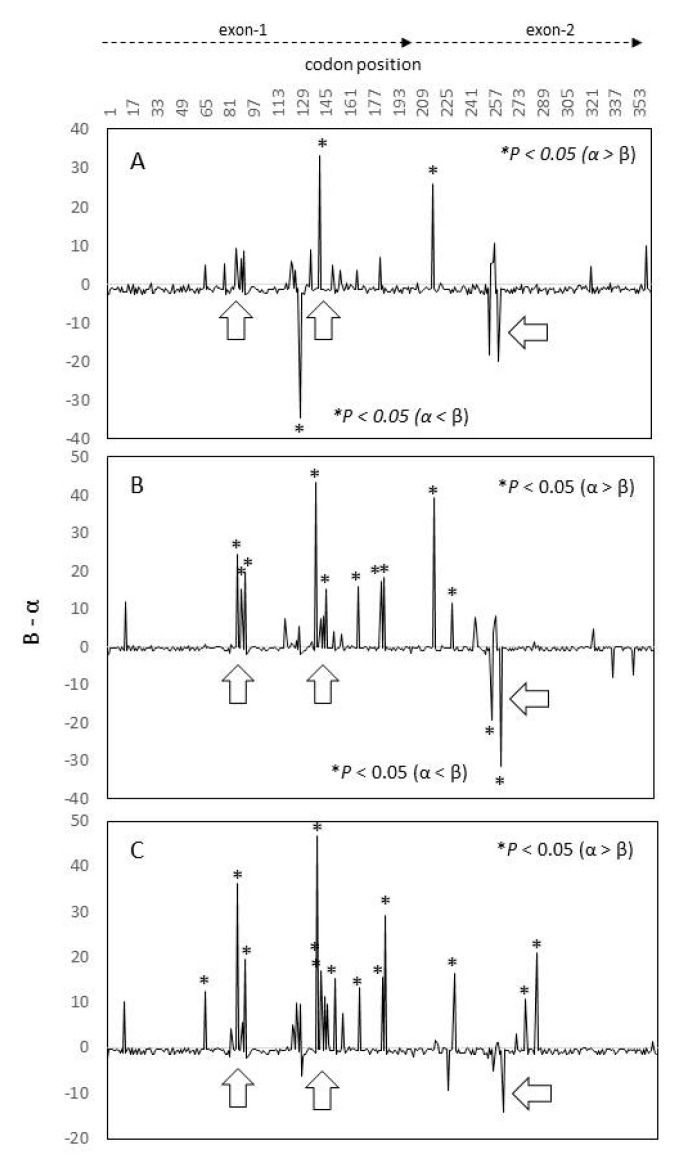
*P. vivax* cyrpa showing codons under selection in parasites from Southern Mexico (**A**), South America (**B**) and Asia (**C**). Codons with values of β-α above or below zero and significant (*) are suggested to be under positive or negative selection, respectively. FUBAR (https://www.datamonkey.org/). Arrows indicate positions under similar selective pressure for all parasites groups.

The *Pvcyrpa* coding gene had an excess of nonsynonymous mutations. The analysis of selection using the differences between rates of nonsynonymous and synonymous changes (β-α) showed that codons with positive values were more frequent in exon-1 than in exon-2 (Figure 3). Parasites from Southern Mexico showed two codons with positive significant values. For South American and Asian parasites several codons had positive β-α values (*P <* 0.05). The Z test of selection shows that the exon-1 sequence had significant positive values in parasites of different origin. These values were higher in parasites from South America or Asia. Meanwhile, the exon-2 sequence showed no deviation from neutrality (Table 5). The MK neutrality index correlated with the previous results, and only exon-1 sequences had significant positive values (Table 5).

### 3.4. Linkage Disequilibrium (LD) and Recombination

**Figure 4:**
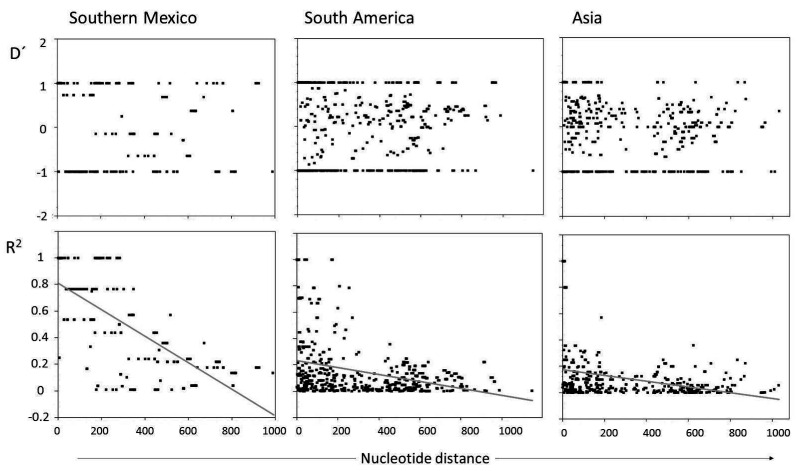
Linkage disequilibrium analysis of the *P. vivax cyrpa* gene. Parasites from Southern Mexico and other regions. The D’ and R^2^ indexes were calculated using only informative sites. For parasites from Southern Mexico, the regression line emerges at an R^2^ value of ~0.8 and decreases steadily whereas for the other parasites, the line initiates at ~0.2 and decays slowly. Most significant values were for nucleotide distances < 300 bp. Exon-1, 1–605nt; intron, 606–828; and exon-2 of 478nt, 823–1306. No gaps were included.

Using only informative segregating sites, of the 190, 561, and 465 pairwise comparisons for parasites from Southern Mexico, South America, and Asia, the numbers of significant comparisons with the Bonferroni correction were 32 (16.8%), 47 (8.3%), and eight (1.7%), respectively. For parasites from Southern Mexico, the D’ index showed most values polarized and few within the +0.5/–0.5 range, while R^2^ values were high, at an average of 0.50 (the ZnS value). The ZnS values were lower for parasites from South America (0.1447) and Asia (0.099). For Southern Mexico, the regression curve crossed the R^2^ axis at ≈ 0.8 and decreased steadily, while the regression line of the South American and Asian parasites crossed the Y axis above and below 0.2, respectively, and decayed slowly (Figure 4). The minimal recombination events for the entire gene sequence in these parasite groups was 3, 9 and 12 for Southern Mexico, South America and Asia, respectively.

### 3.5. Prediction of Polymorphic Residues Potentially Participating in the B-Cell Epitope

**Figure 5:**
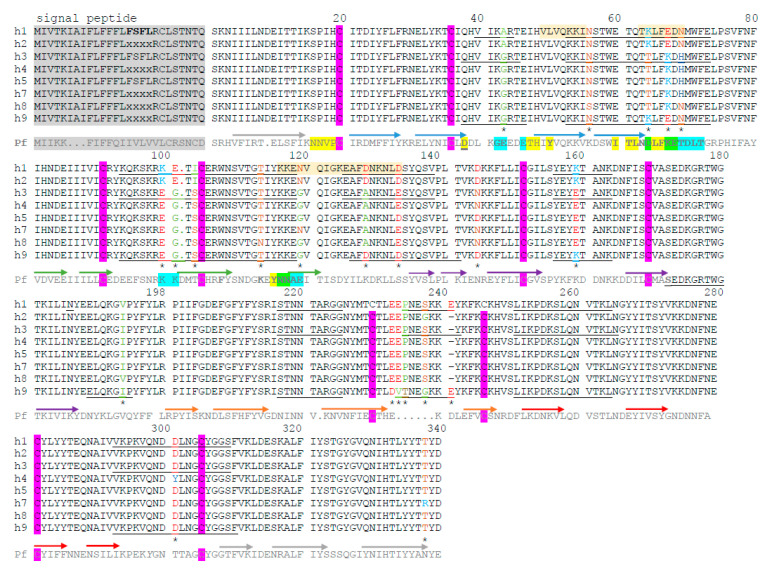
Amino acid variation in *P. vivax* CyRPA from the Southern Mexican isolates. Haplotypes h1-h9 are shown. The conserved residues of the amino acid cysteine are marked with a purple box. The underlined peptide segments had a score above 0.50, resolved using BepiPred lineal epitope prediction 2.0, and are likely to participate in B-cell epitopes. Amino acid changes are indicated by color according to their characteristics. In red, D (Asp) and E (Glu), both polar negatively charged amino acids; in bright blue, K (Lys) and R (Arg), both positively-charged polar amino acids. Brown and light green indicate polar and non-polar amino acids, respectively. FSFL conserved residues were detected in all the sequences obtained in this study, and genomic sequences lacked information from this segment (PlasmoDB). Haplotypes h5 and h6 had a similar amino acid sequence. Pf indicates the *P. falciparum* orthologous sequence, in which the strands of β sheets are indicated using arrows and a different color for each sheet—yellow [4] and blue [20] indicate residues found to be participating in conformational B epitopes by different studies; green indicates residues suggested by both studies [4,20]. Pf D66 is a residue that is relevant for antibody binding to a conformational epitope [20]. Dashes indicate an absence of sequence and dots indicate indels. * indicate amino acid changes among Southern Mexican isolates.

Figure 5 shows the CyRPA amino acid variation among sequences from the Southern Mexican parasites. Important amino acid polymorphism was detected in regions predicted to contain residues participating in B-cell epitopes, and accessible to the protein surface using BepiPred-2.0. These peptide regions coincided with the amino acid residues detected as participating in the CyRPA B-cell epitope of *P. falciparum* [8,34]. The amino acid position was considered without the signal peptide, as suggested by Chen et al., [35]. At 42 amino acid, haplotypes h1 and h2 had Ala while the other haplotypes had Gly. Haplotypes h1, h2 and h9 differed from the other haplotypes at 64 and 67 amino acids—a polar-charged positive Lys vs. a polar not-charged Thr, and a negatively-charged Glu vs. a positively-charged Lys, respectively. At the 100, 101, and 103 residues, both h1 and h2 expressed Lys-Glu-Ile while the other haplotypes had a different Glu-Gly-Ser sequence. At the 119, 128, 144, 159 residues, the variations were Asn vs. Gly, Asp vs. Ala, Asp vs. Asn, and Lys vs. Glu, respectively. The h9 haplotype showed unique significant changes—Glu233Asp, Glu234Val, Pro235Thr, and Ser238Gly—compared to other sequences, and had the codon insert at 241Glu, present in haplotype h1 (Figure 5). The PvCyRPA sequences from the South American and Asian parasites showed similar amino acid substitutions in the predicted B-cell epitopes (Table S2).

## 4. Discussion

The cysteine-rich protective antigen (CyRPA) of *P. vivax* seems to be essential for the parasite’s life cycle and an alternative ligand for reticulocyte invasion [10]. Significant protein polymorphism and a high positive selection pressure acting on this protein could limit its use as a vaccine component or as a serological marker. *P. vivax cyrpa* from Southern Mexico and from other locations showed that mutations and recombination have contributed to generating nucleotide and haplotype diversity. The similarity in the nucleotide diversity pattern across the coding gene suggests that similar evolutionary forces act on *P. vivax cyrpa* parasites of different origin. The phylogenetic trees suggest a weak population structure of *P. vivax* using *pvcyrpa*. The coding gene shows nonsynonymous mutation accumulation, mainly in exon-1. *Pvcyrpa* exon-1 had positive and significant values for the TjD and/or D* and F* (Table 4), selection values (β-α) (Figure 3), Z (HA = dN > dS), and MK tests (Table 5), which suggest that this exon might be under positive balancing selection pressure. Peptide regions that have a significant amino acid variation in exon-1, at a broader level than that in exon-2, are predicted to participate in B-cell epitopes. The coded protein segment from exon-1 seem to be more exposed to the surface than that from exon-2. The latter seems to be under structural constraints.

Unlike *P. falciparum*, both *P. knowlesi* and *P. vivax* lack the RH5 gene. Pkcyrpa, as a single protein, showed itself to be essential for parasite survival, and probably uses a non-BSG requisite for erythrocyte invasion [10]. In *P. vivax*, as in *P. knowlesi*, PvCyRPA is transcribed during schizogony. However, its role in *P. vivax* development has not been determined. Knuepfer et al. [10] showed that the development of *P. vivax* blood stages *in vitro* was significantly reduced by anti-Basigin antibodies in some parasite isolates. Targeting molecules important for the parasite’s life cycle might be limited by their antigenic polymorphism or low immunogenicity. In *P. falciparum,* CyRPA showed limited polymorphism and poor antibody responses in individuals after parasite exposure [3] even though the recombinant PfCyRPA showed itself to be highly immunogenic [4].

The lower diversity found in *P. vivax cyrpa* from Southern Mexico, compared to areas with higher transmission intensity, resembles other genetic markers [34,36,37] and genomic studies [19]. In this study, the nucleotide diversity at *pvcyrpa* was higher (π = 0.0086 ± 0.0005), especially in exon-1 (π = 0.0124 ± 0.00086), than in other microneme protein segments involved in reticulocyte invasion, e.g., *pvdbp_II_* (π = 0.003) and *pvama1_I-II_* (π = 0.007) [34]. PvDBP and PvAMA1 proteins participate in merozoite invasion via the Duffy antigen receptor for chemokines (DARC) [38]. Those microneme proteins showed lower nucleotide diversity than the *pvmsp1_42_* surface protein [34,39], which is important when merozoites first come into contact with reticulocytes. The level of polymorphism in blood-stage antigens seems to be associated with the degree of exposure to the immune system [33]. In PNG, it was reported that PvCyRPA induced IgG-specific antibodies in children, and low levels were associated with protection from clinical disease; thus, antibody levels against this protein might be considered as a marker of concurrent and past exposure to *P. vivax* [11]. Merozoite proteins, such as PvCyRPA, that induce antibody responses, can be good markers of recent exposure and/or protective immunity [35]; nonetheless, this awaits further investigation.

In our study, exon-1 showed an excess of nonsynonymous mutations in parasites of different origin. The positive and significant values of the TjD and other selection tests (β-α values, Z (HA = dN > dS) and MK tests) suggest that *pvcyrpa* exon-1 might be under positive balancing selection pressure in Southern Mexico and other geographic sites [40]. However, the high and significant TjD values by themselves might reflect parasite population contraction. In Southern Mexico, a gradual reduction in malaria cases occurred over the sample collection period (2000–2009, and one sequence from 2015), along with a loss of haplotypes, probably due to genetic drift. Which caused reduction in the nucleotide diversity and this is consistent with the high LD and the few Rm detected, when compared to parasites from South America and Asia. The two most frequent *pvcyrpa* haplotypes (h1/h2) in Southern Mexico were genetically closely related with no further diversification detected, while one divergent haplotype was present in isolate M760A. Furthermore, *P. vivax* hypnozoites can be a confounding factor, and some persistent haplotypes might have corresponded to relapsing parasites [41].

On the other hand, demographic changes in *Plasmodium* populations might affect genome-wide sequences, while natural selection acts on specific segments of the genome, e.g., drug-resistant genes and antigenic molecules, among others [42,43]. *Pvcyrpa* shares characteristics with other merozoite genes that participate in reticulocyte invasion. The sequence comprises semiconserved segments adjacent to highly variable hotspots that accumulate nonsynonymous mutations, likely involved in immune evasion [34]. The h3–h8 haplotypes made up a group of “balanced” haplotypes that were detected across time. In previous studies, *pvama1_I-II_* (but not *pvdbp_II_* or *pvmsp1_42_*) was reported to be under balancing selection pressure in parasites from Southern Mexico in 2006–2007 [34]. To test the hypothesis that Tajima’s D increased as malaria cases in Southern Mexico decreased, the *pvama-1_I-II_* sequences from a longitudinal study were analyzed. In the 1990s, a high transmission and control phase, the TjD was positive (2.072; *P* < 0.05), and these values remained positive for the 2000–2007 period (of lower transmission). The TjD values decreased in sequences from 2008–2011 (Figure S3) [19,34]. Additionally, positive selection scanning in the *P. vivax* genome from the China-Myanmar border detected signatures of positive selection acting on *pvcyrpa* and other blood-stage antigens [43]. In this study, it is suggested that the Asian population is under positive selection pressure (as shown in the Z (HA = dN > dS) and MK tests); however, it is advised that further studies are conducted on a larger sample size to elucidate if correspond to positive balancing signature. The Asian group comprised only a few *pvcyrpa* sequences from Thailand, PNG, and China.

This study represents a first insight into PvCyRPA amino acid polymorphism in residues that potentially participate in linear or conformational B-cell epitopes (Figure 5; Table S2). The presence of protective conformational epitopes has been suggested in *P. falciparum* PfCyRPA [8,34]. In CyRPA, cysteines are largely conserved among *Plasmodium* species (including *P. vivax*) and contribute to the canonical six-bladed β–propeller fold [8]. Alignment of proteins from different species suggests a similar structure but further studies might elucidate whether similar peptide regions are exposed to the protein surface, and can induce blocking and protective antibodies. Protein residues of a protective conformational B-cell epitope were located in *P. falciparum* [35] on similar peptide segments participating in PvCyRPA B-cell epitopes using the BepiPred method. These peptide segments demonstrate an important amino acid polymorphism in *P. vivax*, likely caused by immune selective pressure. Further experimental studies are necessary to identify protective linear and conformational B-cell epitopes in PvCyRPA and in clinical protection, the impact of amino acid variation in immune evasion, and/or peptide regions that are valuable as serological markers.

Interestingly, the *pvcyrpa* intron distinguished the h1 from the h2 haplotype (defined by the coding gene), which might indicate differences in the evolutionary history of the parasites. Introns are not under selective pressure so the limited parasite recombination between some haplotypes has probably been driven by vector restriction. In Southern Mexico, the two main *P. vivax* populations (North American and Central/South American) converged, likely transmitted by different subgenera, namely the *Anopheles* or *Nyssorhynchus* species [33].

## 5. Conclusions

The degree of nucleotide and haplotype diversity was associated to the low transmission intensity in Southern Mexico. Regardless of the parasite’s geographic origin, the pattern of nucleotide diversity across the coding region was similar; this indicates that the structural and/or functional properties are consistent. The results suggest that polymorphism at *pvcyrpa* exon-1 is generated by mutation and recombination, and is probably maintained by positive balancing selection, which might represent an evolutionary advantage to the parasite. Amino acid variation was present at peptide regions potentially participating in B-cell epitopes, which supports the idea that this molecule is under selective immune pressure. A characteristic of malaria blood stage antigens is their participation in merozoite invasion and/or immune evasion. Immunogenicity studies and molecular modeling are required to determine the importance of PvCyRPA as a vaccine candidate and/or a serological marker.

## Figures and Tables

**Table 1:** PCR primers used for the diagnosis and amplification of the *pvcyrpa* gene.

Primer	Sequence (5′–3′)	Specificity	Reference
Reverse primer *UNR*	GACGGTATCTGATCGTCTTC	Universal	[15]
Forward primer *PLF*	AGTGTGTATCCAATCGAGTTTC	*Plasmodium*	[15]
Reverse primer *VIR*	AGGACTTCCAAGCCGAAGC	*P. vivax*	[15]
CYRPA_R1	AGTTGGGATGTGCTACTGGAG	*P. vivax*	This article
CYRPA_F1	TAAGTCTGCTTTCCTCTCTTGGG	*P. vivax*	This article
CYRPA_R2	AGACTGGAAAGACGCAACGG	*P. vivax*	This article
CYRPA_F2	TTGGAGGGACTTGTCCGGTT	*P. vivax*	This article
CYRPA_R3	TGCTCTGTGTAGTAGAGG	*P. vivax*	This article
CYRPA_F3	TTTTTCTCCCCTTGGGAGGCTAC	*P. vivax*	This article
CYRPA_R4	GTGGAAAGAAGTGTGTGGAGGT	*P. vivax*	This article
CYRPA_F4	TATGGGACTTTTGATGGTTG	*P. vivax*	This article
CYRPA_R5	AACTGACTGGTATGAGTCC	*P. vivax*	This article

**Table 2:** Gene and amino acid variation of the *P. vivax cyrpa* coding region among Southern Mexican parasites.

Haplotype	*n*	Exon-1															Exon-2								
		Codon Number/Amino acid Residue																							
		**69**	**82**	**91**	**93**	**95**	**126**	**127**	**129**	**132**	**139**	**145**	**154**	**159**	**170**	**185**	**220**	**258**	**259**	**260**	**261**	**264**	**271**	**326**	**363**
		Ala	Asn	Lys	Glu	Asn	Lys	Glu	Ile	Arg	Thr	Asn	Asp	Asp	Asp	Lys	Val	Leu^1^	Glu	Glu	Pro	Ser	-	Asp	Thr
		Nucleotides																							
h1	5+1	gcg	aac	Aag	gaa	Aat	aaa	gaa	atc	cgg	Acc	aac	gac	gac	Gac	aaa	gtc	cta	gaa	gag	ccg	agc	-	gat	acg
h2	2 + 3	…	…	…	…	…	…	…	…	…	…	…	…	…	…	…	a..	…	…	…	…	g.t	gaa	…	…
h3	3 + 1	.g.	…	.c.	a..	c..	g..	.g.	.g.	..c	…	gg.	.c.	..g	a..	g..	a..	…	…	…	…	…	-	…	…
h4	2 + 0	.g.	…	.c.	a..	c..	g..	.g.	.g.	..c	…	gg.	.c.	..g	a..	g..	a..	…	…	…	…	…	-	t..	…
h5	0 + 1	.g.	…	.c.	a..	c..	g..	.g.	.g.	…	…	gg.	.c.	…	…	g..	a..	…	…	…	…	…	-	…	…
h6	0 + 1	.g.	…	.c.	a..	c..	g..	.g.	.g.	..c	…	gg.	.c.	…	…	g..	a..	…	…	…	…	…	-	…	…
h7	1 + 0	.g.	…	.c.	a..	c..	g..	.g.	.g.	..c	…	…	.c.	..g	a..	g..	a..	…	…	…	…	…	-	…	.g.
h8	1 + 0	.g.	.g.	.c.	a..	c..	g..	.g.	.g.	..c	.a.	gg.	.c.	..g	a..	g..	…	…	…	…	…	…	-	…	…
h9	1 + 0	.g.	…	…	…	…	g..	.g.	.g.	..c	…	gg.	…	…	…	…	a..	..t	..c	.t.	a..	g.t	gaa	…	…
		Amino acid change:																							
		Gly	Ser	Thr	Lys	His	Glu	Gly	Ser	*	Asn	Gly	Ala	Glu	Asn	Glu	Ile	*	Asp	Val	Thr	Gly	Glu	Tyr	Arg

All seven sequences obtained in this study had codons ctc ttc tcc ttc at the amino end, similar to the Sal I sequence (X_001615090.1); the codons are numbered to this sequence. * No amino acid variation. N, number of isolates (sequences from PlasmoDB + from this study). (-) Absence of codon. Codons from 69 to 220 and from 258 to 363 correspond to exon-1 and exon-2, respectively. Note that most frequent haplotypes were present in our study and found in the PlasmoDB repository [19].

**Table 3:** Diversity parameters of the *P. vivax cyrpa* coding gene in Southern Mexican parasites, and in isolates from other geographic regions.

Diversity	*Entire Coding Gene*: 1083 bp	exon-1 (605 bp)	exon-2 (478 bp)
Southern Mexico (*n* = 22)			
SS	25	16	9
M	25	16	9
H	9	7	6
Hd ± SD	0.861 ± 0.044	0.697 ± 0.0068	0.797 ± 0.046
π ± DE	0.0086 ± 0.0005	0.0124 ± 0.00086	0.004 ± 0.00084
θw ± (DE nr, fr)	0.0063± (0.0024, 0.0012)	0.00727 (0.00294, 0.00182)	0.00517 (0.0023, 0.00172)
Rm	4	3	0
South America (*n* = 45)			
SS	39	25	14
M	41	27	14
H	27	20	14
Hd ± SD	0.975 ± 0.009	0.92 ± 0.027	0.910 ± 0.018
π ± DE	0.01228 ± 0.00041	0.01467 ± 0.00055	0.00925 ± 0.00043
θw ± (DE nr, fr)	0.00824 (0.00263, 0.00132)	0.00945 (0.00321, 0.00189)	0.00670 (0.00254, 0.00179)
Rm	11	8	2
Asia (*n* = 19)			
SS	40	25	15
M	43	28	15
H	18	16	15
Hd ± SD	0.994 ± 0.019	0.982 ± 0.022	0.971 ± 0.027
π ± DE	0.0138 ± 0.00070	0.01627 ± 0.0010	0.01067 ± 0.00076
θw ± (DE nr, fr)	0.01057 (0.00392, 0.00167)	0.0118 (0.000021; 0.0023)	0.00898 (0.00375, 0.00232)
Rm	12	8	3

SS, segregating sites; M, mutations; H, haplotype; Hd, haplotype diversity; Rm, minimal number of recombination events. InDels were not included in the analysis.

**Table 4:** Neutrality tests for the *P. vivax cyrpa* coding sequence; entire sequence and by exon.

Parameters	Southern Mexico			South America			Asia		
	Coding Gene	exon-1	exon-2	Coding Gene	exon-1	exon-2	Coding Gene	exon-1	exon-2
Syn	3	1	2	5	1	4	5	1	4
Nonsyn	22	15	7	32	22	10	31	20 ^3^	11
Tajima’s D (TjD)	1.358	2.493 ^1^	−0.713	1.573	1.657	1.178	0.917	0.993	0.704
TjD (syn)	0.638	1.553	−0.174	0.674	0.242	0.688	0.246	−0.035	0.305
TjD (nonsyn)	1.401	2.425 ^1^	−0.805	1.652	1.697	1.210	1.003	1.040	0.787
Fu & Li’D*	0.042	0.870	−1.284	1.3043 ^2^	1.496 ^1^	0.599	0.912	1.443 ^1^	−0.157
Fu & Li’F*	0.513	1.5765 ^2^	−1.297	1.6120 ^2^	1.763 ^1^	0.935	1.045	1.492 ^2^	0.106

syn, synonymous mutations; nonsyn, nonsynonymous mutations. *cyrpa*, 1083 bp; exon-1, 605 bp; exon-2, 478 bp. *P* < 0.05 ^1^; 0.10 > *P* > 0.05 ^2^. Twenty-one changes ^3^.

**Table 5:** Selection tests for the *P. vivax cyrpa* coding gene among different parasite groups.

Geographic Origin	Gene Fragment	Polymorphic Changes within *P. vivax* Groups		Z-Test (dN > dS)	*P*-Value	Fixed Differences between Species *		McDonald and Kreitman (Neutrality Index)	*P*-Value
		Synonymous	Nonsynonymous			Synonymous	Nonsynonymous		
Southern Mexico:									
	Coding region	2	21	2.399	0.009	82	84	10.25	<0.001
*n* = 22	exon-1	1	15	2.101	0.0189	48	56	12.857	0.0020
	exon-2	1	6	0.875	0.1912	33	27	7.333	0.0544
South America:									
	Coding region	4	32	2.8205	0.0028	81	82	7.902	<0.001
*n* = 45	exon-1	1	23	3.479	<0.001	48	56	19.714	<0.001
	exon-2	3	9	0.3909	0.3483	32	25	3.840	0.06238
Asia:									
	Coding region	4	30	2.883	0.0023	81	84	7.232	<0.001
*n* = 19	exon-1	1	20	3.529	<0.001	48	56	17.143	<0.001
	exon-2	3	10	0.803	0.2116	32	27	3.951	0.0647

Fisher’s exact test * *P*-value (two tailed). * XM_004221254.1 *Plasmodium cynomolgi* strain B hypothetical protein (PCYB_053730) mRNA; gaps were not included in the analysis, adjustment to 1071 bp exons 1–2. Exon-2 showed that mutations had no deviation from neutrality. The Z test was run in MEGA v6 and the MK neutrality index in DnaSP v6. Under neutrality, the ratio of replacement to synonymous fixed substitutions (differences) between species should be the same as the ratio of replacement to synonymous polymorphisms within species.

## Data Availability

Data is contained within the article or Supplementary Materials.

## Supplementary Materials

The following are available online at https://www.mdpi.com/2073-4425/12/1/29/s1, Figure S1. Geographic origin of isolates from Southern Mexico. Figure S2. Global ML phylogenetic tree of the *P. vivax cyrpa* coding gene. Figure S3. Tajima’s D (TjD) values using *pvama1* sequences from Southern Mexico. Table S1. *Pvcyrpa* gene sequences extracted from PlasmoDB and used in this study. Table S2. Description of *Pvcyrpa* genetic and amino acid polymorphism in global sequences.
